# Renoprotective effects of cinnamon oil against APAP-Induced nephrotoxicity by ameliorating oxidative stress, apoptosis and inflammation in rats

**DOI:** 10.1016/j.jsps.2021.01.002

**Published:** 2021-01-23

**Authors:** Saeed Alshahrani, Mohammad Ashafaq, Sohail Hussain, Manal Mohammed, Muhammad Sultan, Abdulmajeed M. Jali, Rahimullah Siddiqui, Fakhrul Islam

**Affiliations:** a Department of Pharmacology and Toxicology, College of Pharmacy, Jazan University, Saudi Arabia; b Substance Abuse Research Center (SARC), College of Pharmacy, Jazan University, Saudi Arabia; c Department of Pharmaceutics, College of Pharmacy, Jazan University, Saudi Arabia

**Keywords:** Acetaminophen, Cinnamon oil, Nephrotoxicity, Oxidative stress, Caspases-3, 9, Inflammation

## Abstract

Acetaminophen (APAP) is used as a primary medication in relieving moderate pain and fever. However, APAP is associated with toxic effects in renal tissue that appear because of its free radicals property. The principle goal of the present work is to assess the kidney damage by APAP and its restore antioxidative property of cinnamon oil (CO). Animals were distributed into six animals each in six groups. Rats were administered with three varying doses of CO from 50 to 200 mg/kg b.w. respectively and only a single dose of APAP. APAP induced an alteration in serum biochemical markers, imbalance in oxidative parameters, morphological changes in kidney tissue along with increased interleukins cytokines (IL-1β & 6) and caspase (3, 9) levels. CO administration significantly ameliorates all the parameters and histopathological changes were restored. Moreover, it also restored the activities of antioxidative enzymes. Our work proved that an variance of oxidative markers in the kidney by APAP is ameliorated by CO in rats. Thus, CO could be used in reducing APAP-induced nephrotoxicity.

## 1 Introduction

Acetaminophen (APAP) was invented in 1889 and is commonly used as a fever and pain relieving agent (Reshi et al., 2020). APAP poisoning is the most common in the world (Gunnell et al., 2000) and its excess dose is mostly related with hepatic and renal damage (Saleem and Iftikhar 2019). Although clinicians believe that APAP is a safe drug with limited adverse effects. Nevertheless, some studies demonstrated that the death of patients, in some cases, could occur even at low doses and that may be due to the higher responsiveness to its toxic effect (Ucheya and Igweh, 2006). While nephrotoxicity is less common than hepato-toxicity caused by APAP overdose, contrary reports have shown that renal degeneration and renal failure could occur even without liver injury (Jones and Vale, 1993).

At clinically prescribed dose, APAP in the liver as well as in the kidney is conjugated with glucuronate and sulfate to produce nontoxic metabolite that are excreted (Pingili et al., 2019). At overdose, the conjugation advanced towards saturation and spurts in oxidative metabolism mediated by cytochrome P450 increases, consequently a reactive transitional metabolite NAPQI generated (Abdul et al., 2008). Toxic NAPQI quickly accompanied by intracellular reduced glutathione (GSH) to form a nontoxic water-soluble mercapturic acid conjugate which is eliminated through the kidney (Reshi et al., 2020). Elevated NAPQI level is resultant in depletion of GSH which produced an imbalance between prooxidant and antioxidant that leads to production of ROS in the kidney (Akgun et al., 2020). Furthermore, APAP transforms into the harmful p-aminophenol a nephrotoxin metabolite in the kidney which initiate tissue damages specifically tubular and cortical necrosis (Salama et al., 2015). Earlier reports also showed that renal necrosis and oxidative stress contribute a major role in nephrotoxicity (Ghosh et al., 2010). Along with APAP-induced oxidative stress and renal damages, further detrimental cascades like, inflammatory and apoptotic mediators are activated which aggravated an advanced injury in the kidney (Ahmad et al., 2012). Activated interleukin IL-1 β and IL-6 enhance oxidative stress that adds further damage to kidney tissue. Also, activation of apoptotic caspase 3 and 9 accelerate extensive oxidative stress and acute kidney damages (Wu et al., 2017).

Researchers suggest that APAP-induced hepatorenal damage is due to oxidative strain and inflammation (Das et al., 2010, Kheradpezhouh et al., 2010). However, drugs or plants that have antioxidant properties might be a potential cure for nephrotoxicity by reducing the oxidative imbalance (Galal et al., 2012, Abdul Hamid et al., 2012, Hussain, 2017). Cinnamon is used in flavoring and as a spice that is regularly used in seasonings, baked goods and chili sauces. CO and its derivatives having antidiabetic, antimicrobial, antioxidant, anti-inflammatory and anticancer activities hence it can be used as food additives. (Tuzcu et al., 2017, Zare et al., 2018, Fabio et al., 2003). Murcia et al. (Murcia et al., 2004) have investigated the antioxidative properties of cinnamon oil (CO) compared with butylated hydroxyl anisole (BHA) (E‑320) a common food antioxidant, research findings showed that the CO demonstrates a higher proportion of inhibition the lipid peroxidation (Morgan et al., 2014). The major constituents of CO are cinnamaldehyde (Gunawardena et al., 2015) and eugenol (Barboza et al., 2018) which have been well documented that they having various therapeutic properties like antidiabetic, antiapoptotic, antioxidative and anti-inflammatory property (Kim and Choung, 2010, Lv et al., 2017). There are scarce or no data reported which examine the nephroprotective activity of the CO in the rat model. Therefore, we hypothesize that CO may offer nephroprotection due to its antioxidant, anti-inflammatory and anti-apoptotic activity in APAP-induced nephrotoxicity. Our group has already reported the beneficial effects of CO against APAP in the liver and brain (Hussain et al., 2020; Ashfaq et al., 2020).

In our work, our group analyzed the beneficial effects of CO against APAP-induced nephrotoxicity. Nephrotoxicity was examined by assessing the renal function marker, lipid peroxidation (LPO) as a marker of free radical presence, antioxidative enzymes along with inflammatory cytokine IL-1β, IL-6, caspase 3, 9 expression and histological changes in kidney tissue.

## 2 Materials and Methods

### 2.1 Chemicals

Acetaminophen (A5000-1 kg), cinnamon oil (C7267-100 ml) and supplementary chemicals were bought from Sigma chemical. Interleukin IL-1β & IL-6 (ab100768 and ab100772) and caspases-3 and 9 (ab39401 and ab65608) kits were purchased from Abcam, UK. Renal function biomarker (Urea- UR 456, Uric acid-UA 230 and Creatinine-CR 510) kits were got from Randox Laboratories Ltd (Crumlin, UK).

### 2.2 Animals

180–220 g male Wistar rats were procured from MRC (Medical Research Center) and housed in the laboratory of SARC (Substance Abuse Research Centre) College of Pharmacy, Jazan University. The procedure was permitted by the Institutional Research Review and Ethics Committee (312/1509/1440, IRREC). The ambient temperature, humidity (45–55%, 25 ± 2℃) and a 12-h dark/light cycle was maintained throughout the experiments. Standard food and water were freely accessible to rats.

### 2.3 Experimental plan

Animals were randomly divided in six groups each having six animals. The control group served with saline (0.9% NaCl) orally once daily for 15 days. The second group received CO (200 mg) orally once for 15 days. The third group was treated with APAP 2 g/kg once orally in saline whereas the fourth, fifth and sixth groups was treated with APAP 2 g/kg once orally in saline before three days of execution of experimental work. Three varying doses of CO (50, 100 and 200 mg/kg) were given in fourth, fifth and sixth group (APAP + CO) once daily for 15. The selections of a dose of APAP and CO were based on literature (Ozatik et al., 2019, Bellassoued et al., 2019).

### 2.4 Collection of sample for biochemical estimation

Rats were anesthetized (diethyl ether) and the blood sample were withdrawn from an ocular puncture after completion of the treatment regime. Blood serum was obtained by centrifuging samples at 2000–3000 rpm for 10 min and stored in a deep freezer for further analysis. The renal marker (creatinine, urea and uric acid) was analyzed by using commercially available test kits. Thereafter, animals were sacrificed by cervical dislocation under light ether anesthesia using glass desiccator as described by Ibrahim et al. (2016) and kidney tissue was dissect out to prepare homogenates and post mitochondrial supernatants (PMS) in 0.1 M Na Phosphate buffer to compared with serum results. LPO and GSH were assayed for oxidative stress along with enzymes superoxide dismutase (SOD) Catalase, glutathione reductase (GR) and glutathione peroxidase (GPx). All biochemical measured by using a double beam UV-spectrophotometer (UV-1800, Shimadzu JAPAN). From each groups, kidney tissues were kept in formalin fixative solution for histopathological studies.

### 2.5 Lipid peroxidation assay

As per Hussain and Ashafaq (2018) method, the LPO level in the kidney homogenate were determined using 10% trichloroacetic acid and 0.67% thiobarbituric acid. Pink colour complex formed with TBA read at 535 nm and levels in LPO nmol/g tissue were determined by a molar extinction coefficient of 1.56 × 10^5^ M^−1^ cm^−1^.

### 2.6 Reduced glutathione determination

Depleted level of kidney GSH was following the method of Hussain et al., (2020). In this assay equal volume of 4% sulfosalicylic acid (SSA) and kidney sample incubated in ice. Colour changes after adding 0.4 ml supernatant, 1.2 ml of sodium phosphate buffer and 0.4 ml of DTNB were measured at 412 nm and shown as GSH μmol/g of tissue.

### 2.7 Superoxide dismutase activity

Ashafaq et al., (2020) assay was used to evaluate SOD activity observing the auto-oxidation of (-)-epinephrine. The sample consists of glycine buffer (50 mM, pH 10.4) and 0.2 ml of PMS. 4.02 × 10^3^ M^−1^ cm^−1 M^ extinction coefficient used to assess SOD activity.

### 2.8 Catalase activity

Kidney catalase activity was assayed according to Khuwaja et al., (2020). The rate of H_2_O_2_ decomposition in 50 μl of kidney sample and 1.95 ml of PB was spectrophotometrically measured as the nmol H_2_O_2_ consumed/min/mg protein at 240 nm.

### 2.9 Assay of glutathione reductase

Waseem et al., (2014) procedure was followed for GR activity. The sample mixture consisted, 0.1 M PB 7.4 pH, 1 mM oxidized GSH 0.5 mM EDTA, kidney sample and 0.1 mM NADPH. The activity of GR was taken at 340 nm and determined using molar extinction coefficient 6.22 × 10^−3^ M^−1^ cm^−1^.

### 2.10 Assay of glutathione peroxidase

Kidney GPx activity was measured according to Waseem et al. (2014). Absorbance of GPx activity recorded at 340 nm and expressed as nmol NADPH oxidized/min/mg protein. The reaction assay consists of 0.05 M PB, 1 mM GSSG, 0.25 mM H_2_O_2_ and 100 μl kidney sample.

### 2.11 Determination of cytokine and caspases in kidney tissue

Cytokines interleukin (IL-1 β, IL-6) levels and Caspase (3, 9) activity analyzed by commercially available ELISA Kit from Abcam (UK). Absorbance were taken at 450 and 405 nm respectively using 96 well plate Reader (ELx 800TM BioTek®, USA). The manufacturer’s scheme was followed for data analysis.

### 2.12 Histopathological assessments

Hematoxylin and eosin (H & E) staining were achieved to examine the normal architecture of cells and cellular irregularity in control and APAP treated groups. H & E staining of kidney tissue were done after several tissue process in formalin and paraffin fixation. Paraffin fixed tissue section (5 µm) were obtained by microtone sectioning. Different gradients of ethanol were used to rehydrate and dehydrate sections during the staining process. Section from the various group was analyzed under a light microscope at 200 × magnification.

### 2.13 Estimation of protein

Protein was approximated as per the Hussain et al., (2020) and bovine serum albumin (BSA) used as reference standard.

### 2.14 Statistical analysis

Data collected from various experimentation were shown as mean ± SEM of six rats. Methods of statistical analysis perform through analysis of variance (ANOVA), followed by Tukey-Kramer’s test. The p < 0.05 was assumed significant.

## 3 Results

### 3.1 Biochemical study

Magnificently increase of renal markers in the APAP treated group as compared with the control. APAP treated with CO group (APAP + CO 50, 100, 200) caused a marked decreased in blood serum levels of urea, uric acid and creatinine as compare with APAP treated rats (table 1) and a similar pattern of results were obtained in tissue homogenates.

**Table 1:** Biochemical effects of cinnamon oil in serum and homogenate of APAP induced nephrotoxicity in rats.

Parameters	Control	CO (100 mg/kg b.w.)	APAP	APAP + CO (mg/kg b.w.)		
				50	100	200
Urea (mg/dl) (Serum)	80 ± 5.71	90 ± 9.52	170.74 ± 11.38^***^	144.07 ± 8.81^#^	110.23 ± 5.25^##^	100.32 ± 0.31^###^
Uric Acid (Serum) (mg/dl)	1.43 ± 0.17	1.35 ± 0.13	2.10 ± 0.21^**^	1.98 ± 0.021	1.90 ± 0.15^#^	1.67 ± 0.32^##^
Uric Acid (Homogenate) (mg/dl)	0.78 ± 0.08	0.83 ± 0.05	2.52 ± 0.10^***^	1.96 ± 0.13^#^	1.61 ± 0.11^###^	1.29 ± 0.15^###^
Creatinine (Serum) (mg/dl)	1.76 ± 0.14	1.69 ± 0.11	6.94 ± 0.23^***^	3.57 ± 0.19^##^	3.30 ± 0.27^##^	3.18 ± 0.10^###^
Creatinine (Homogenate) (mg/dl)	1.40 ± 0.17	1.57 ± 0.19	3.87 ± 0.25^***^	1.72 ± 0.22^###^	1.49 ± 0.12^###^	1.30 ± 0.16^###^

Estimation of serum biomarker in APAP induce nephrotoxicity in rats. CO treatment significantly reversed the serum markers in APAP + CO group as compared to the APAP group. Values are expressed as mean ± S.E.M of n = 6 animals. Significance was determined by one-way ANOVA followed by Tukey-Kramer post-hoc test for multiple comparisons.

**P < 0.01, ***p < 0.001 APAP vs. control.

^#^*p* < 0.05, *^##^p* < 0.01, *^###^p* < 0.001 APAP + CO vs. APAP.

### 3.2 Effect of CO on LPO

**Fig. 1:**
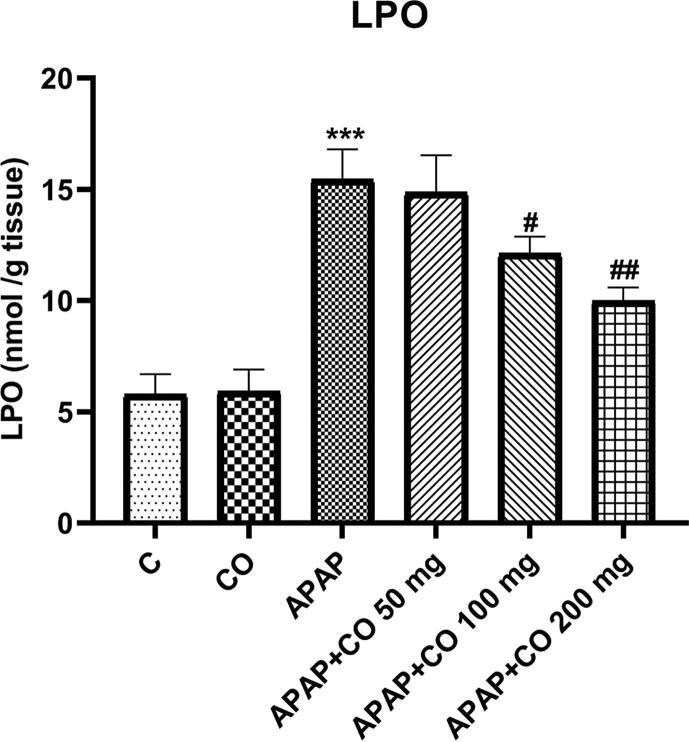
Effect of CO on kidney tissue levels of LPO in nephrotoxicity induced by APAP. Data presented as Mean ± SEM (n = 6). ***p < 0.001 designates significant difference between only APAP with control group, *^##^*p < 0.01 and *^#^*p < 0.05 shows significant difference from APAP untreated group (VEH).

Significant elevation of LPO was examined in the only acetaminophen group. CO ameliorates the elevated level of LPO and it was significant with APAP + CO 100 (p < 0.05) and APAP + CO 200 (p < 0.01) of CO as shown in Fig. 1.

### 3.3 CO treatment elevated GSH and activities of antioxidative enzymes in kidney

CO treatment significantly elevates GSH level and ameliorates antioxidative enzymes (SOD, CAT, GR and GPx) in APAP + CO 100 and 200 groups when compared to the APAP group. The level of GSH and activity of all enzymes were diminished in the APAP group markedly as compared with the control group. (Table 2).

**Table 2:** Activities of antioxidant enzymes (SOD, CAT, GR and GPx) in kidney tissue.

Parameters	Control	CO	APAP	APAP + CO 50	APAP + CO 100	PAP + CO 200
**GSH** (µmol GSH/ g tissue)	13.75 ± 1.18	14.27 ± 1.35 (3.78%)	7.15 ± 0.87** (-48.00%)^a^	7.86 ± 0.98 (9.93%)^b^	8.90 ± 0.84# (24.47%)^b^	11.71 ± 0.81^##^ (63.77.12%)^b^
**SOD** (nmol of Epinephrine protected from oxidation/min/mg protein)	45.17 ± 2.54	45.63 ± 3.11 (1.01%)	23.28 ± 2.65*** (-48.46%)^a^	24.64 ± 1.78 (5.84%)^b^	29.81 ± 1.89 (28.04%)^b^	34.95 ± 1.76^##^ (50.12%)^b^
**CAT** (nmol of H_2_O_2_ consumed/min/mg/protein)	12.67 ± 0.67	13.01 ± 1.01 (2.68%)	5.61 ± 0.34*** (-55.72%)^a^	6.96 ± 0.76 (24.06%)^b^	8.38 ± 0.41# (49.37%)^b^	9.52 ± 0.87^##^ (69.69%)^b^
**GR** (nmol of NADPH oxidized/min/mg protein)	21.38 ± 1.24	21.91 ± 1.77 (2.47%)	8.05 ± 0.79*** (-62.38%)^a^	9.57 ± 0.52 (18.88%)^b^	10.92 ± 1.12 (35.65%)^b^	16.73 ± 0.95^##^ (51.88%)^b^
**GPx** (nmol of NADPH oxidized/min/mg protein)	18.79 ± 0.87	19.42 ± 1.96 (3.35%)	7.41 ± 0.47*** (-60.56%)^a^	9.70 ± 1.143 (30.90%)^b^	11.55 ± 1.32^##^ (55.87%)^b^	12.29 ± 1.08^##^ (65.85%)^b^

APAP induce significant alterations in GSH level and the activities of antioxidant enzymes (SOD, CAT, GR and GPx) in APAP group as compared to the control group. CO treatment significantly protected the activity of these enzymes dose dependently in APAP + CO group as compared to the APAP group. Values in parentheses show the percentage increase or decrease with respect to their control. Values are expressed as mean ± S.E.M of n = 6 animals. Significance was determined by one-way ANOVA followed by Tukey-Kramer post-hoc test for multiple comparisons.

a Values in parentheses indicate the percentage change vs. Control.

b Values in parentheses indicate the percentage change vs. APAP.

** *p* < 0.01;

*** *p* < 0.001 APAP vs. control.

# *p* < 0.05, *^##^p* < 0.01, *^###^p* < 0.001 APAP + CO vs. APAP.

### 3.4 Assay of CO on cytokine

**Fig. 2 and 3:**
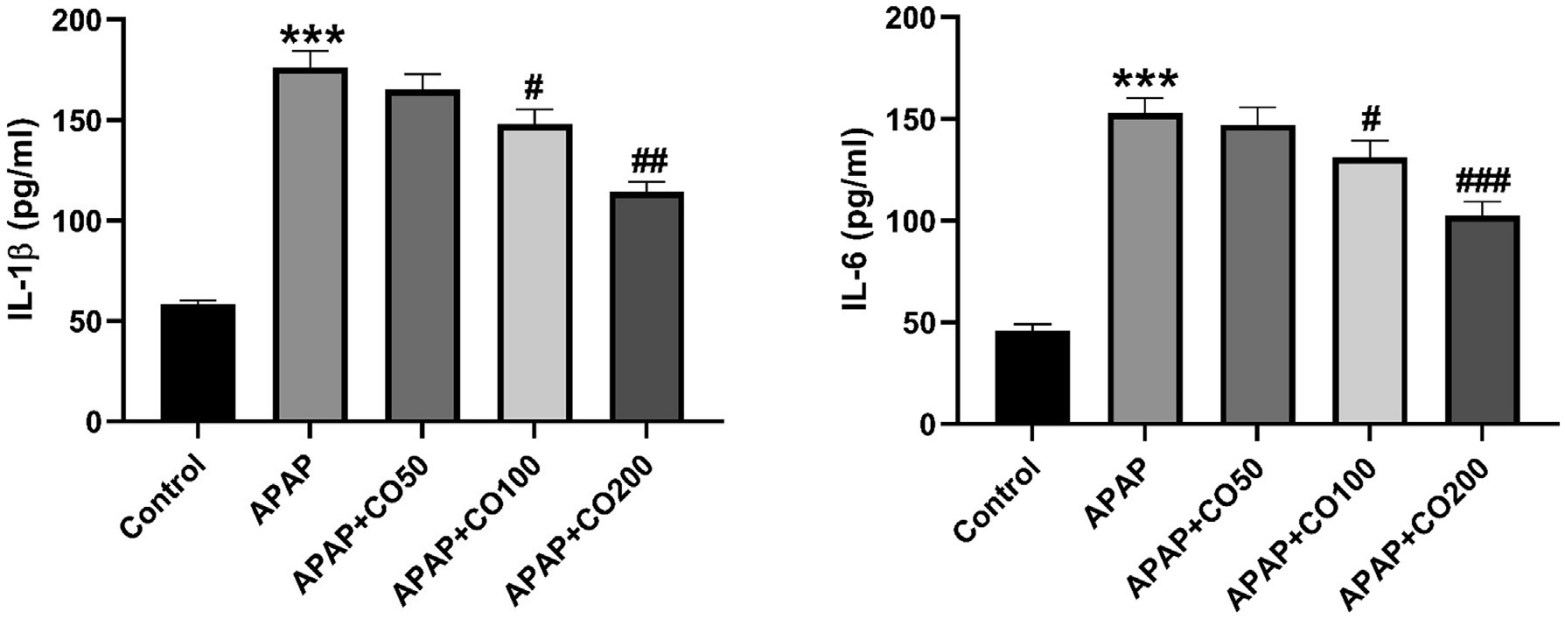
CO attenuates APAP induced activation of inflammatory mediators IL-1β (3) and IL-6 (4) in the kidneys of rats treated with APAP. ELISA results are presented as group Mean ± SEM (n = 6). ***p < 0.001 compared to control group; *^#^*p < 0.05 ^##^p < 0.01and ^###^p < 0.001, respectively, compared to the APAP group.

APAP induced an increase in expression of cytokines (IL-1β, IL-6) in the APAP group. Moreover, CO treatment magnificently attenuated the expression of cytokines in the APAP + CO 100 and 200 groups (Fig. 2 and Fig. 3).

### 3.5 CO affects caspase 3 and 9

**Fig. 4 and 5:**
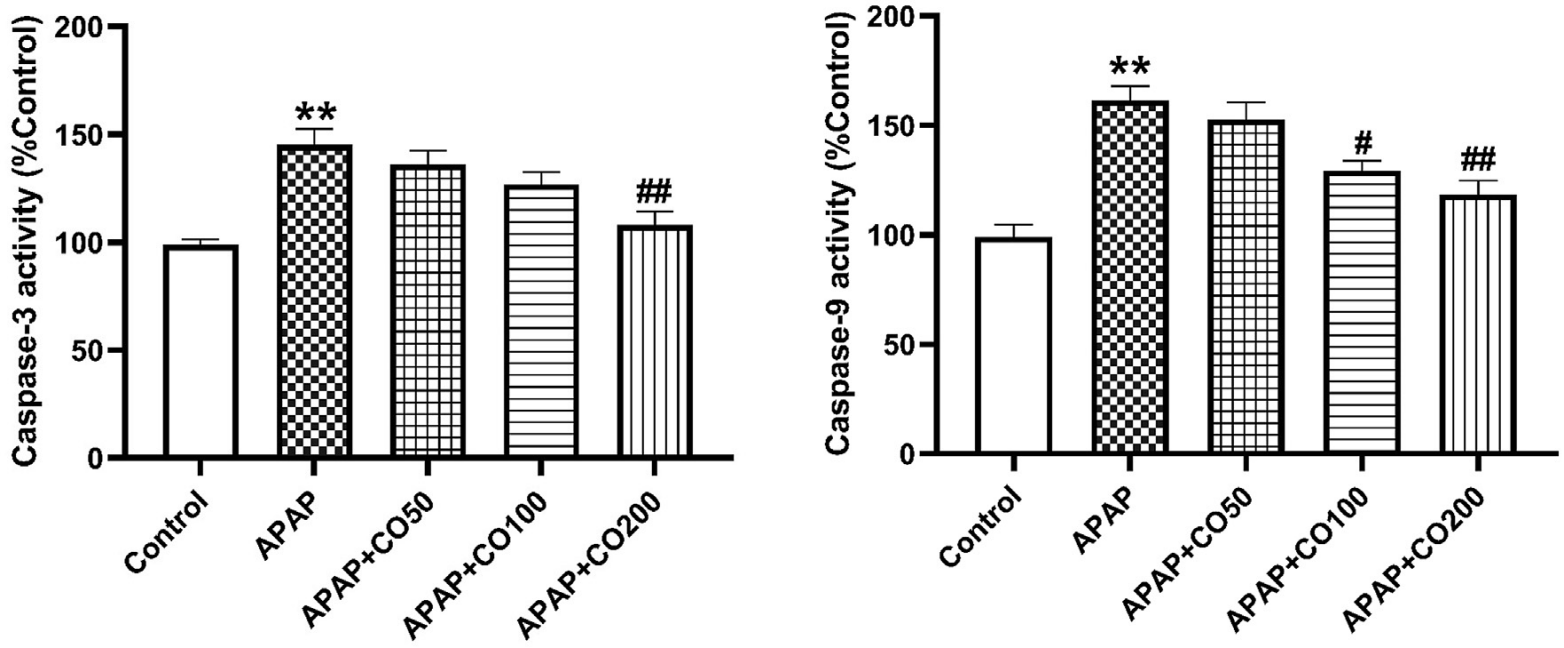
CO attenuated activation of caspase-3 (5) and caspase-9 (6) in kidney tissue of rats treated with APAP. Data presented as Mean ± SEM (n = 6). **p < 0.01 designates significant difference between only APAP from control group, *^#^*p < 0.05 and ^##^p < 0.01 shows significant difference from APAP untreated group (VEH).

Expression of caspases was increased significantly in APAP treated animals. The levels of Caspase 3 and 9 decreased remarkably when treated with CO in APAP + CO 100 and 200 groups (Fig. 4 and 5).

### 3.6 Effect on kidney histology

**Fig. 6:**
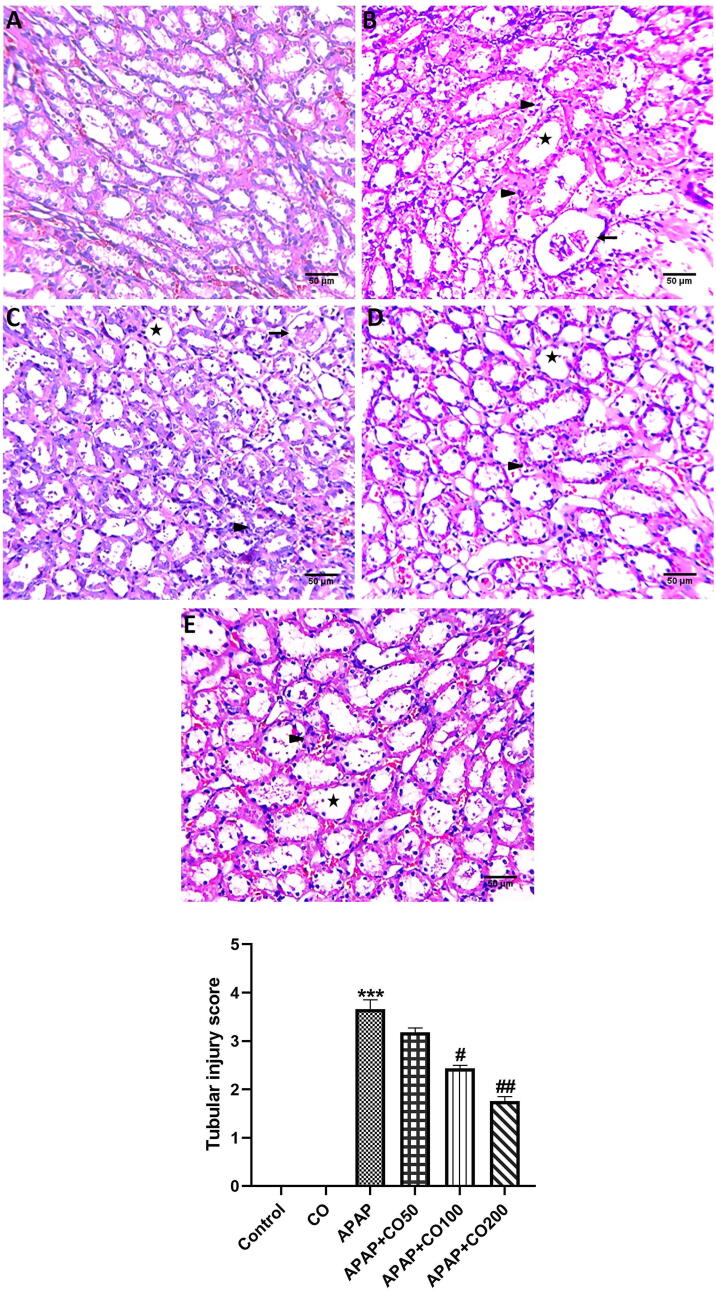
Effect of CO on morphological changes in APAP treatment rat using H&E staining in the kidney of control, APAP and different doses of APAP + CO groups. Kidneys from the control group (A) showed no histological changes. Pathological alteration included infiltrating cells and tubular necrosis (arrowhead), epithelial degeneration (arrow), tubular dilatation (star), and swelling in APAP treated rats (B). Histological damages were ameliorated in kidneys from rats treated with CO in APAP + CO (C, D and E) when compared with kidneys in rats treated with APAP alone. Values are expressed in means ± S.E.M. (n = 6). ***P < 0.001 APAP vs. control group; ^#^P < 0.05, ^##^P < 0.01 APAP + CO vs. APAP alone-treated group.

In APAP and CO treated rats, histopathology changes in rat kidney were examined. Our assessments specify that APAP treatment showed noticeable structural changes of the kidney, categorized by extensive cellular damage or necrosis and the presence of inflammatory cell infiltration with tubular dilation. Nuclei were often hyperchromatic and pyknotic. The cellular architect was asymmetrical in form and presented deviations in nuclear/cytoplasmic ratio. However, CO treatment protected cellular injury in APAP + CO as compared to the APAP group alone (Fig. 6 panel A and B).

## 4 Discussion

According to the USA, FDA the pharmaceutical ingredients of APAP in advised drugs need to not be > 325 mg, the maximum prescribed dose of APAP to < 4.0 g for an adult (Mitka 2014). Now a day APAP is used in inducing nephrotoxicity in experimental models. According to the clinical and animal findings, N-deacetylase, cytochrome *P*-450 pathway and prostaglandin are some potential mechanisms of nephrotoxicity (Bessems and Vermeulen, 2001). The renal aggregation of a toxic metabolite of APAP could be leading to a series of biochemical responses that culminate in kidney damage (Schnellman, 2001). With an overdose of APAP, the reactive metabolite produced by the P-450-dependent metabolism, N-acetyl-P-benzoquinone imine could not be scavenged by reduced GSH levels resulting in kidney failure (Karakus et al., 2013). Reports of Karadeniz (Karadeniz et al. 2008) and Ajami (Ajami et al. 2010) shows an increase in free radicals production changes the filtration area and coefficient, so these factors might reduce the filtration in the glomerular resulting in accumulation of urea, uric acid and creatinine in the serum and tissue homogenate (uric acid and creatinine).

An elevated level of LPO leads to tissue damage (Reshi et al., 2020). SOD, GSH, CAT, GR and GPx are the main antioxidative enzymes to neutralize reactive molecules or protect against kidney oxidative damage (Whidden et al., 2011). APAP in toxic dose elicited a magnificent rise in the level of renal LPO with a significant reduction in SOD, GSH and catalase. Our results are also supported by a previous finding (Canayakin et al., 2016, Ashafaq et al., 2020) Renal injury leads to decreased in enzyme activity and aggregation of free radical superoxide results in kidney damage. Co-treatment of CO significantly reduced the renal toxicity caused by APAP. The protective activities CO may be through ameliorating effect on antioxidative and its anti-inflammatory markers. Our study indicates that co-treatment with CO shows a significant amelioration in the LPO caused by APAP in the form of reduction of LPO level, accompanied by increased activities of all enzymes in the APAP + CO group.

APAP mediated kidney toxicity also encourages inflammatory cytokine interleukin (IL-1β, 6). There is growing evidence to show that inflammation is also accountable for the APAP induced pathogenesis in kidney tissue (Cermik et al., 2013). Moreover, oxidative stress provokes inflammation is more aggravating to kidney pathogenesis. Activated inflammatory cytokine triggers macrophages which are releasing more reactive radicals responsible for oxidative stress. In this study, we observe that APAP treated group showed a significant increase in interleukins (IL-1β, 6) level as compared with control. However CO treatment significantly subsides the increased level of IL-1β and IL-6 in the APAP + CO treated group. Our findings are inconsistent with earlier reports, where APAP induced IL-1β and IL-6 level was significantly attenuated by administration of polyphenolic compound possess anti-inflammatory activity (Hussain et al., 2020, Kandemir et al., 2017). APAP induced-kidney damage may provoke caspase dependent apoptotic cascade in an animal model (Ghosh et al., 2010). Oxidative damages may alter the membrane potential which is responsible for the upregulation of caspase (3, 9) expression in damaged kidneys. We observed upregulated caspases in APAP treated group. CO administration significantly suppressed the overexpression of caspases in the APAP + CO group when correlated with only APAP group. These findings are well documented in other reports where treatment with polyphenolic compounds possess antioxidant and antiapoptotic activity decreases overexpression of caspases in APAP induced kidney damage (Ahmad et al., 2012).

Histopathological observations also confirmed that the APAP exhibit noteworthy cellular changes representing tubular and necrotic damage in the kidney whereas CO reverses these cellular adjustments and protected the kidney in CO treated groups. This study also explores that APAP recruits tubular damage by inducing necrosis or cell death as reported earlier (Raskovic et al., 2018). Nevertheless, CO treatment against APAP represents betterment in cellular architect owing to robust antioxidant activity. In contrast with our examination, recent studies also representing naturally occurring antioxidant compounds fortified damage in the kidney (Raskovic et al., 2018, Koca-Caliskan et al., 2018).

## 5 Conclusion

Current finding indicates that APAP-induced nephrotoxicity facilitated through oxidative stress inflammation and overexpression of caspase-3 and 9. Antioxidative and anti-inflammatory are the main mechanism of action by which CO show beneficial effect CO against APAP induced nephrotoxicity. Further experimentation is necessary to explore nutraceutical application in a clinical trial.

## Declaration of Competing Interest

The authors declare that they have no known competing financial interests or personal relationships that could have appeared to influence the work reported in this paper.
